# Pathology in the Age of Artificial Intelligence (AI): Redefining Roles and Responsibilities for Tomorrow's Practitioners

**DOI:** 10.7759/cureus.56040

**Published:** 2024-03-12

**Authors:** FNU Sandeep, NFN Kiran, Zubair Rahaman, Pooja Devi, Ahmed Bendari

**Affiliations:** 1 Pathology, Mayo Clinic, Rochester, USA; 2 Pathology, Northwell Health, New York, USA; 3 Internal Medicine, University at Buffalo Jacobs School of Medicine and Biomedical Sciences, Buffalo, USA; 4 Pathology/Hematopathology, University of Pennsylvania Health System, Philadelphia, USA; 5 Pathology, Northwell/Lenox Hill Hospital, NewYork, USA

**Keywords:** ai integration, digital pathology, ai in pathology, artificial inteligence, pathology

## Abstract

The evolution of pathology from its rudimentary beginnings around 1700 BC to the present day has been marked by profound advancement in understanding and diagnosing diseases. This journey, from the earliest dissections to the modern era of histochemical analysis, sets the stage for the next transformative leap to the integration of artificial intelligence (AI) in pathology. Recent research highlights AI’s significant potential to revolutionize healthcare within the next decade, with a particular impact on diagnostic processes. A majority of pathologists foresee AI becoming a cornerstone in diagnostic workflow, driven by the advent of image-based algorithms and computational pathology. These innovations promise to enhance the precision of disease diagnosis, particularly in complex cases, such as cancers, by offering detailed insights into the molecular and cellular mechanisms. Moreover, AI-assisted tools are improving the efficiency and accuracy of histological analysis by automating the evaluation of immunohistochemical biomarkers and tissue architecture. This shift not only accelerates diagnostic processes but also facilitates early disease management, crucial for improving patient outcomes. Furthermore, AI is reshaping educational paradigms in pathology, offering interactive learning environments that promise to enrich the training of future pathologists. Despite these advancements, the integration of AI in pathology raises ethical considerations regarding patient consent and data privacy. As pathology embarks on this AI-augmented era, it is imperative to navigate these challenges thoughtfully, ensuring that AI enhances rather than replaces the pathologist’s role. This editorial discussed the historical progression of pathology, the current impact of AI on diagnostic practices, and the ethical implications of its adoption, underscoring the need for a symbiotic relationship between pathologists and AI to unlock the full potential of healthcare.

## Editorial

Pathology, the scientific study of diseases and their processes, has fascinated humanity since records of disease manifestations date back to as early as 1700 BC. This editorial explores the evaluation of pathology, highlighting how humans have utilized an array of remarkable techniques through the centuries to delve into the causes of disease - knowledge that is intrinsically linked to the development of treatments. From the early, somewhat mysterious human dissections in Ancient Alexandria to intricate single-cell sequencing and in situ hybridization of tissues seen today, the field of pathology has undeniably progressed significantly. Yet, the journey does not end here. Similar to other scientific fields, pathology will continue to advance over time, propelled by ongoing technological innovations.

Research has anticipated that artificial intelligence is one of the most significant technological advancements that will change the future of healthcare in the next 10 years [1,2]. AI has a potential for generativity, and several early trials have already shown promising results in its potential implementation. In fact, a study clearly states that around 80% of pathologists believe that AI will become an integral part of diagnostic workflows in the coming few years [3]. Pathology in the age of AI has seen a sharp rise in its progress, particularly due to the use of image-based algorithms that help streamline the whole process. Additionally, the use of computational pathology and the implementation of AI tools is also responsible for the paradigm shift in how pathology services are managed. Ultimately, all these transformative changes are responsible for meeting the needs of the modern science of precision medicine.

Most of the time, clinical manifestations of the diseases are not enough to rule out the primary diagnosis of the overall pathological process. Management of complex diseases, such as cancer, does not solely rely on the diagnosis. Numerous factors need to be unfolded to start the correct treatment regimen. For example, the treatment of a grade 1 tumor would not be the same as that of a metastatic carcinoma. In such circumstances, pathology is useful in giving a final answer by uncovering the underlying molecular and cellular mechanisms. Unfortunately, due to the rising number and complexity of diseases, diagnosing every single one becomes arduous for pathologists. That's why the integration of AI-assisted diagnostic tools extends far beyond the scope of digital pathology alone, playing a pivotal role in enhancing diagnostic accuracy. Recent studies over the last five years have robustly demonstrated that AI can analyze whole slide images (WSI) to provide diagnoses that are on par with those of experienced pathologists [4]. Beyond image analysis, AI's capabilities span a diverse array of applications, including both not limited to the precise quantification of immunohistochemical biomarkers, cell enumeration, and examination of various tissue characteristics such as the spatial arrangement of cells, density and distribution patterns, and overall tissue architecture [1].

Furthermore, AI technologies encompass a wide range of additional functionalities critical to modern pathology practice. These include quality control measures, normalization processes, digital speech recognition for efficient data entry, and organization of data into synoptic reports. Large language models further contribute to this expansion, offering sophisticated tools for data analysis and interpretation. Collectively, these advancements underscore the multifaceted utility of AI in revolutionizing diagnostic processes, highlighting the technology’s importance in both augmenting and streamlining pathological assessments.

From a practical standpoint of daily clinical care, pathologists typically rely on a limited number of slides and images to determine the histological type and grade of the disease. Additionally, whether the pathologist's staging is accurate depends on their practical skills, meaning for the proper prediction, an opinion from a senior pathologist is a must. Furthermore, the integration of complex environmental factors and morphological patterns in a prognostic index is a challenging task for the pathologist. However, AI-assisted tools are much more well-acquainted with gathering information and resulting in the particular stage of the disease [5]. Additionally, these tools are also capable of visualizing the response of a particular treatment on tissue framework, revolutionizing everything the pathology has done so far [5].

Furthermore, the outcome of the disease can drastically change if the management is started in the early stages of the disease process. However, appropriate and timely patient management often needs an efficient pathology workflow. This can be made possible using several AI tools capable of not only improving the speed of the overall process but also helping in making the most accurate diagnosis. Pathologists rely on a chain of events involving a thorough assessment of the slides, which is a time-consuming and error-prone process. Remarkably, AI tools can streamline the process and reduce the range of errors that can occur while analyzing scanned slides [1,5]. Additionally, these tools can sort the cases based on the priority level. The cases that need urgent evaluation are given top priority and are analyzed first compared to the elective cases, ultimately increasing the efficiency of the pathology workflow [1,5].

The outlook of pathology in the age of AI is much more adaptable than simply its role in the diagnosis and management of complex disease pathology. AI can provide specific interactive functions to create an excellent teaching environment for trainees. By providing a perfect teaching environment, AI ensures the maximum engagement of the next generation of pathologists, providing a better chance for them to acquire expert skills during the overall process. Therefore, better involvement of the pathologists in learning helps them acquire better skills. Ultimately, there is no second thought that the integration of AI in pathology is reshaping the roles and responsibilities of pathologists. Therefore, there is a need for a transformative shift in how diagnostic tasks are approached, validated, interpreted, and reported.

Traditionally, pathologists remain the front liners of disease diagnosis, relying on their knowledge, experience, and intuition to interpret complex histological patterns and make informed diagnostic decisions. However, times have changed, and AI-assisted tools are better equipped to recognize disease-related characteristics. But this doesn’t mean that AI would take the central role of pathologists, ultimately increasing the automation of routine tasks. Instead of just displacing pathologists, AI will augment the pathologist's role, growing a symbiotic relationship between man and machine. Using AI for the initial slide screening and decision support, pathologists can speed up the overall process and give more time to complex and challenging cases. There is still a lot to explore, and scientists are not entirely sure what the future holds for the pathology workforce. However, it is confirmed that the collaborative efforts of pathologists and AI will lead to the development of novel diagnostic algorithms, predictive models, and therapeutic strategies in the upcoming few years.

AI is becoming an important part of pathology and its associated process, and there is no stopping to that. However, there is a need to address the ethical implications of using AI in developing pathology tools by collecting patient-related information. Critical ethical considerations include patient consent, transparency, accountability, and equity in access to AI-assisted diagnostic services, along with ensuring that the credit and benefits derived from Al tools are distributed fairly. The developers of the AI tools should obtain informed consent from the patients and the public before using their data to validate their tools. Additionally, patients should have the authority over the amount of information they would like to share with the developers. This would help build their trust over time, and hopefully, people will be able to trust AI-assisted tools as they trust pathologists in the upcoming few years.

In conclusion, prior to the advent of modern pathology marked by technical advancement in microscopy and staining, as pioneered by the early giants of the field such as Papanicolaou and Virchow, the treatment of disease was often a matter of conjecture, based on rudimentary tissue examination. Advancements in pathology have since allowed for a nuanced understanding of disease processes at the microscopic level, enhancing treatment strategies. With the integration of AI, we are now witnessing a transformative era in pathology that demands adaptability from pathologists to realize the full potential of healthcare in diagnosing and treating disease effectively.
